# Cross-reactivity of IgM anti-modified protein antibodies in rheumatoid arthritis despite limited mutational load

**DOI:** 10.1186/s13075-021-02609-5

**Published:** 2021-09-03

**Authors:** Sanne Reijm, Theresa Kissel, Gerrie Stoeken-Rijsbergen, Linda M. Slot, Corrie M. Wortel, Hugo J. van Dooren, Nivine E. W. Levarht, Arieke S. B. Kampstra, Veerle F. A. M. Derksen, Pleuni Ooijevaar-de Heer, Holger Bang, Jan W. Drijfhout, Leendert A. Trouw, Tom W. J. Huizinga, Theo Rispens, Hans U. Scherer, René E. M. Toes

**Affiliations:** 1 Department of Rheumatology, Leiden University Medical Center, Leiden, The Netherlands; 2 Sanquin Research and Landsteiner Laboratory, Academic Medical Center, Amsterdam, The Netherlands; 3 Orgentec Diagnostika, Mainz, Germany; 4 Department of Immunology, Leiden University Medical Center, Leiden, The Netherlands

**Keywords:** Rheumatoid arthritis, AMPA, IgM, Somatic hypermutation, B cells

## Abstract

**Background:**

Anti-modified protein antibodies (AMPA) targeting citrullinated, acetylated and/or carbamylated self-antigens are hallmarks of rheumatoid arthritis (RA). Although AMPA-IgG cross-reactivity to multiple post-translational modifications (PTMs) is evident, it is unknown whether the first responding B cells, expressing IgM, display similar characteristics or if cross-reactivity is crucially dependent on somatic hypermutation (SHM). We now studied the reactivity of (germline) AMPA-IgM to further understand the breach of B cell tolerance and to identify if cross-reactivity depends on extensive SHM. Moreover, we investigated whether AMPA-IgM can efficiently recruit immune effector mechanisms.

**Methods:**

Polyclonal AMPA-IgM were isolated from RA patients and assessed for cross-reactivity towards PTM antigens. AMPA-IgM B cell receptor sequences were obtained by single cell isolation using antigen-specific tetramers. Subsequently, pentameric monoclonal AMPA-IgM, their germline counterparts and monomeric IgG variants were generated. The antibodies were analysed on a panel of PTM antigens and tested for complement activation.

**Results:**

Pentameric monoclonal and polyclonal AMPA-IgM displayed cross-reactivity to multiple antigens and different PTMs. PTM antigen recognition was still present, although reduced, after reverting the IgM into germline. Valency of AMPA-IgM was crucial for antigen recognition as PTM-reactivity significantly decreased when AMPA-IgM were expressed as IgG. Furthermore, AMPA-IgM was 15- to 30-fold more potent in complement-activation compared to AMPA-IgG.

**Conclusions:**

We provide first evidence that AMPA-IgM are cross-reactive towards different PTMs, indicating that PTM (cross-)reactivity is not confined to IgG and does not necessarily depend on extensive somatic hypermutation. Moreover, our data indicate that a diverse set of PTM antigens could be involved in the initial tolerance breach in RA and suggest that AMPA-IgM can induce complement-activation and thereby inflammation.

**Supplementary Information:**

The online version contains supplementary material available at 10.1186/s13075-021-02609-5.

## Background

The presence of autoantibodies is an immunological hallmark of rheumatoid arthritis (RA). About 50–70% of RA patients harbour anti-citrullinated protein antibodies (ACPA), directed against citrullinated proteins generated by the conversion of arginine to citrulline [1]. ACPA recognize a wide spectrum of antigens and are found in different isotypes [2–4]. More recently, autoantibodies towards other post-translational modifications (PTMs) have been described in RA. Anti-carbamylated protein antibodies (ACarPA) and anti-acetylated protein antibodies (AAPA) are present in approximately 50% and 40% of RA patients, respectively [5, 6]. Carbamylation is a non-enzymatic process, resulting from the modification of a lysine-residue into homocitrulline. Acetylation is mediated by acetyltransferases and leads to a conversion of lysine-residues into acetyllysines. ACPA, ACarPA and AAPA were initially considered as separate autoantibody responses, because the targeted modifications are distinct and located at different positions in the protein. However, these autoantibodies, collectively called anti-modified protein antibodies (AMPA), often concur in RA [6, 7]. Recently, it has been shown, at the polyclonal- and monoclonal level, that AMPA-IgG can be highly cross-reactive towards all three PTMs, providing the molecular underpinnings for their simultaneous presence in patients [8–12]. Likewise, B cells expressing citrullinated protein-reactive IgG B cell receptors (BCR) can be activated by carbamylated and/or acetylated antigens [10]. Moreover, mice immunized with a carbamylated or an acetylated protein develop AMPA responses directed towards both PTMs [13]. Together, these data indicate that a cross-reactive AMPA-IgG response is present and that IgG+ B cells recognizing citrullinated antigens can be triggered by the exposure to different types of PTMs.

Similarly, it has been described that ACPA-IgM recognize several citrullinated antigens on the polyclonal level [14–16]. However, it is currently unclear whether this broad reactivity results from the presence of polyclonal ACPA-IgM recognizing distinct citrullinated antigens or whether cross-reactive monoclonal ACPA-IgM are the main players underlying these observations. Likewise, it remains to be determined whether ACPA-IgM, like ACPA-IgG, are cross-reactive towards other PTMs, such as homocitrulline and acetyllysine. Investigating the reactivity patterns of monoclonal AMPA-IgM is relevant to understand the extent to which PTM antigens can be recognized by (naïve) AMPA-expressing B cells. Insight into these matters is important to better understand the nature of antigens that could initiate the RA-specific AMPA response.

Furthermore, it remains unknown whether the emergence of autoreactivity through the recognition of PTM-self-antigens relies on somatic hypermutations (SHM). Interestingly, ACPA-IgG have undergone extensive SHM, carrying on average 50 IGHV nucleotide mutations [11, 17, 18]. This could be instrumental in generating PTM-reactivity as the introduction of autoreactivity via SHM has been described for other diseases [19–21]. Indeed, loss of citrulline reactivity has been reported for a predicted germline ACPA-IgG obtained after over 35 ‘back’ mutations to generate ‘ancestral’ antibody sequences [11, 22, 23]. Likewise, it has been suggested that SHM could explain the cross-reactive nature of ACPA-IgG by showing that somatic mutations can confer binding to multiple citrullinated epitopes [22]. As AMPA-IgM presumably undergo limited SHM, they are an ideal autoantibody response to study germline responses. Therefore, we analysed the antigen recognition profile of polyclonal and monoclonal AMPA-IgM and generated monoclonal germline AMPA-IgM. Furthermore, since ACPA-IgG are known to activate complement, we determined the relative effectiveness of complement activation by AMPA-IgM to evaluate whether AMPA-IgM, as first isotype produced in an antibody response, could potentially initiate inflammation [24]. The results will help us to gain further understanding of the potential triggers of AMPA responses and to understand the breach of tolerance of AMPA expressing B cells in RA.

## Material and methods

### Patient material

Peripherial blood samples were obtained from RA patients visiting the outpatient clinic of the Rheumatology department of the Leiden University Medical Center. All RA-patients were RF-IgM positive and showed high ACPA levels (> 340 U/ml) or detectable AAPA levels from the IMPROVED clinical trial [15].

### Single cell sorting and BCR sequencing

PBMCs were isolated using Ficoll-Paque and stained with Live/dead Fixable Aqua dead cell stain kit 405 nm (life technologies), CD3-PB (UCHT1, BD), CD14-PB (M5E2, BD), CD19-APC-Cy7 (SJ25C1, BD), CD20-AF700 (2H7, Biolegend) and CD27-PE-Cy7(M-T271, BD). Antigen-specific staining was performed using CCP2-BV605, CCP2-APC and CArgP2-PE, or HC55-APC and HC55-PE [25] or TT-APC and TT-PE. Antigen-specific single B cell sorts were performed on a BD FACSAria III 4 L cell sorter at the Flow cytometry Core Facility (FCF) of the Leiden University Medical Center (LUMC) in Leiden, Netherlands. The cells were cultured on irradiated CD40L L cells in complex RMPI medium for 10–12 days [26]. RNA isolation, cDNA production, ARTISAN PCR and sequencing was performed as previously described [27, 28].

### IgM expression vectors and monoclonal antibody production

Codon optimized AMPA heavy chain (HC) and light chain (LC) variable (V)-gene sequences, coupled to the IGHM or the IGLC3/IGKC constant regions (UniProt), respectively, the Kozak sequence and the HVAT20 leader peptide were designed in GeneArt (Life Technologies) and ordered at IDT. The constructs, as well as the codon-optiomized J-chain (UniProt) used for the generation of pentameric molecules, were cloned into a pcDNA3.1 (+) expression vector. The IgM HC, LC and J-chain constructs were transfected into Freestyle^TM^ 293-F cells (Gibco) using Opti-MEM® (Gibco) medium and 293Fectin (Thermo Fisher). The supernatants were harvested 5–6 days post transfection. IgG1 monoclonal antibodies (mAbs) with identical V-genes were cloned, produced and purified as previously described [10] .

### Protein modification, peptide synthesis, antigen labelling and antibody purification

Experimental procedures for protein modification, peptide synthesis, antigen labelling and antibody purification are given in the supplementary materials and methods. Peptide sequences are given in Table S2.

### Germline reversion of IgM sequences

Variable (V), diversity (D) and joining (J) genes were identified by alignment with the respective germline sequences of the IMGT reference directory that shows the highest identity using IMGT/V-QUEST [29] (Table S1). The ‘Germline without CDR3’ sequences were generated by reverting mutations into germline while retaining the original CDR3 sequence. The ‘Germline with CDR3’ sequences were obtained by reverting the complete sequence into germline including mutations within the CDR3 region, except for junctions and deoxynucleotidyl transferase (TdT) N nucleotides. Sequences with multiple amino acid changes were orderd at IDT as desribed above. Single amino acid replacements were performed with the Q5 Site-Directed Mutagenesis Kit (New England Biolabs) according to the manufacturer’s instructions. Primers were designed using NEBaseChanger v1.2.9. All mutations were confirmed by sequencing.

### Enzyme-linked immunosorbent assays (ELISAs)

Peptide-, protein- and cross-inhibition ELISAs were performed as previously described [5, 10]. The antibody and antigen concentrations are mentioned in the respective figure legends. Different concentrations for distinct antigens were used for antigen coating to achieve optimal ELISA readouts. AMPA-IgM binding was detected using a HRP-conjugated Goat-anti-human IgM secondary antibody (Millipore, AP114P).

For the Tetanus Toxoid (TT) ELISA, 1.5 LF/ml TT was coated to Nunc Maxisorp plates (Thermo Scientific) and incubated overnight at RT. After 1 h blocking in coating buffer (0.1 M Na_2_CO_3_ 0.1 M NaHCO_3_ in MQ) + 1%BSA at 37 °C, samples were incubated for 2 h at 37 °C. Monoclonal IgM binding was detected using a HRP-conjugated Goat-anti-human IgM secondary antibody (Millipore, AP114P).

For the complement ELISAs, CCP2/CHcitP2 (1 μg/ml) or cit-vimentin 59-74 (10 μg/ml) were coated on streptavidin plates (Microcoat, #65001). All steps were performed for 1 h at 37 °C. Monoclonal antibodies were diluted in PBT (PBS + 1%BSA + 0,05%Tween) and added to the plate. C-active normal human serum (NHS) in GVB++ (gelatin veronal buffer containing Ca^2+^ and Mg^2+^) buffer was added as a source of all complement components to allow C3c-deposition. EDTA, which chelates Ca^2+^ and Mg^2+^ and thereby inhibits complement activation, was added as a negative control [24]. Binding was detected using a rabbit anti-human C3c (DAKO, A0062) primary and a goat anti-rabbit-HRP secondary antibody (DAKO, P0448). Absorbance of all ELISAs was measured at 415 nm using ABTS and H_2_O_2_.

### Western blot

Western blot analysis was performed as previously described [5]. In brief, PTM-Fibrinogen proteins (Fibrinogen, Cit-, CarP- and Ac-Fibrinogen) were subjected to 4–15% SDS-polyacrylamide gels (Bio Rad). Subsequently, immunoblotting was performed on a Nitrocellulose membrane (Bio Rad). Blots were incubated in 3% skim milk powder/PBS/0.05%tween (PTE) for 1 h at RT. Following washing with PBS/0.05% tween (PT), the blots were incubated at 4 °C overnight with 2 μg/ml monoclonal AMPA-IgM diluted in 5 ml PTE. After washing with PT, blots were incubated for 1 h at RT with a HRP-conjugated goat-anti-human IgM secondary antibody (Millipore, AP114P), diluted 1:5000 in 5 ml PTE. Blots were washed and bound antibodies visualized using enhanced chemiluminescence (GE Healthcare, RPN2109).

## Results

### Polyclonal ACPA-IgM are cross-reactive towards homocitrullinated and acetylated antigens

**Fig. 1:**
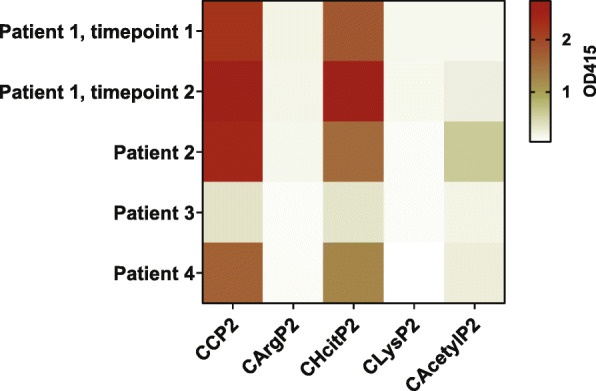
Cross-reactivity of polyclonal ACPA IgM. Heatmap of PTM-Cyclic Peptide 2 IgM ELISA of CCP2 isolated plasma samples from 5 different samples of 4 ACPA+ RA patients. Isolated CCP2 binding antibodies from 5 ml plasma were diluted 10 to 40 times

Previously, it has been shown that polyclonal ACPA-IgM recognize several citrullinated antigens [16]. To determine whether ACPA-IgM are also cross-reactive towards other PTMs and can thus be called AMPAs, we isolated polyclonal ACPA using a CCP2-column from 5 different samples from RA patients. Subsequently, a IgM specific ELISA was performed and the isolated ACPA-IgM tested for binding towards one antigen in three different modifications (citrulline, homocitrulline and acetyllysine) and their respective control (arginine or lysine) (Fig. 1). Mixing experiments, including mixture of CCP2-IgG+ plasmas with RF-IgM+ plasmas as control, showed no signal in the ACPA-IgM ELISA. These results indicate that ACPA-IgM-reactivity was not explained by the presence of RF-IgM (Figure S1). ACPA-IgM from all RA patients showed cross-reactivity towards homocitrulline whereas ACPA-IgM from patients 2 and 4 was also cross-reactive towards acetyllysine.

### AMPA-IgM expressing B cells can be detected in peripheral blood of RA patients and have undergone limited somatic hypermutation

To investigate the AMPA-IgM response in more depth, we single cell isolated AMPA-IgM expressing B cells of 4 RA patients using citrullinated- or acetylated-peptide tetramers. Like ACPA-IgG expressing B cells, the AMPA-IgM-expressing B cells were CD20+CD27+, indicating that they are part of the memory B cell compartment [30]. The gating strategy is shown in Figure S2. As a control, a tetanus-specific IgM expressing B cell was isolated. After 10 days of culture, 4 out of 9 single B cell culture supernatants, including the tetanus-specific IgM B cell culture, contained antibodies reactive towards the antigen used for B cell isolation and not towards its unmodified counterpart as detected by ELISA (Figure S3A). Two of the AMPA-IgM expressing B cells (2D5 and 1G8) were isolated with a citrullinated peptide (CCP2) whereas the third B cell (1E3) was isolated with an acetylated vimentin peptide (HC55). After culture, the variable domain of the BCR was sequenced and aligned to the IMGT database to determine V(D)J usage and the number of mutations. The isolated ACPA- or AAPA-IgM expressing B cells had undergone limited SHM as the BCR contained 3–16 immunoglobulin heavy chain variable region (IGHV) mutations (Table 1). This is considerably lower than for ACPA-IgG, which contain on average 50 IGHV mutations [17]. The amount of IGHV mutations of the IgM-AMPA B cells was similar to the IGHV mutations of the anti-TT IgM, indicating that the extensive SHM of ACPA-IgG is obtained after class switch recombination. Next, we used the obtained sequences to recombinantly generate pentameric monoclonal AMPA-IgM. The integrity of the produced mAbs was confirmed using analytical size exclusion chromatography (SEC) and native-PAGE (Figure S3B-C). As indicated by an early elution from the SEC column and by the high apparent molecular weight in the native gel, our results show that we were able to generate pentameric human AMPA-IgM molecules (Figure S3C) and that the mAbs were reactive towards the antigen used for isolation (Figure S3A).

**Table 1:** Characteristics of the single cell sorted AMPA-IgM expressing B cell sequences. IGHVDJ, IGLVJ and number of IGHV mutations were determined by alligment to the IMGT database

	2D5	1G8	1E3	Anti-TT
Antigen for isolation	CCP2	CCP2	Acetylated-vimentin (HC55)	Tetanus Toxoid
Patient	5	5	6	7
Heavy chain	μ	μ	μ	μ
IGHV	V7-4-1	V7-4-1	V4-59	V5-10-1
IGHD	D4-17	D7-27	D5-24	D3-10
IGHJ	J6	J4	J6	J3
IGH-CD3 aa	CARGTHDYADYYYYGMDVW	CARGTEDGDAYFDYW	CARHIQSLVTTGYYTYPMDVW	CARSYYGPGNYDAFDIW
#IGHV mutations nt	3	7	16	16
Light chain	λ	λ	κ	κ
IGLV	V10-54	V4-69	V2-28	V1-9
IGLJ	J3	J2	J2	J4
IGL-CDR3aa	CSAWDNSLSAWVF	CQTWGTDTVVF	CMQALQNPRAF	CQQLHSYPRTF
#IGLV mutations nt	4	8	7	9

### Monoclonal AMPA-IgM are cross-reactive towards different PTM antigens

**Fig. 2:**
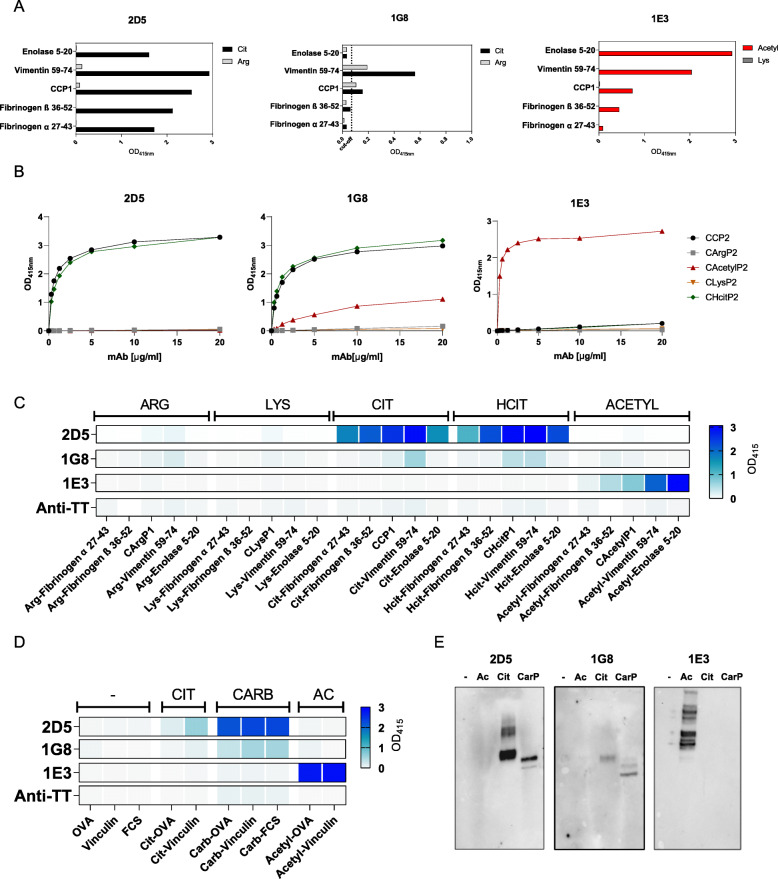
Cross-reactivity of monoclonal AMPA-IgM. **A** ELISA with 10 μg/ml mAb on different citrullinated peptides and arginine-control peptides for 2D5 and 1G8 or with different acetylated peptides and lysine-control peptides for 1E3 (coated 10 μg/ml antigen). The cut-off for positivity was based on the anti-TT IgM control mAb. **B** Titration ELISA of the mAbs 2D5, 1G8 and 1E3 on PTM-Cyclic Peptide 2 (coated 1 μg/ml antigen). **C** Heatmap of a PTM antigen ELISA with 10 μg/ml monoclonal AMPA-IgMs 2D5, 1G8 and 1E3 and anti-TT-IgM control mAb on different peptides (coated 10 μg/ml antigen) and **D** proteins (coated 10 μg/ml antigen). Binding is represented by the optical density at 415 nm. **E** Western blot of monoclonal AMPA-IgMs 2D5, 1G8 and 1E3 (2 μg/ml) on PTM-Fibrinogen and unmodified fibrinogen (0.25–2 μg)

To investigate whether the AMPA-IgM mAbs were restrictive to the antigen used for isolation or display a broader reactivity as observed for AMPA-IgG, we investigated whether 2D5 and 1G8, both obtained from B cells isolated with CCP2, could recognize other citrullinated antigens as well. As depicted in Fig. 2A, both antibodies, although more prominently for 2D5, were specifically reactive towards multiple citrullinated antigens and did not recognize the arginine counterparts. Likewise, 1E3, obtained from a B cell isolated with an acetylated vimentin peptide (HC55), displayed reactivity to a broad range of acetylated peptides, but not to the lysine control variant (Fig. 2A). These results indicate that the ability of ACPA and AAPA to recognize several citrullinated, respectively, acetylated antigens is not confined to IgG, but already present within the IgM repertoire.

We next determined whether this broad reactivity is restricted to the same PTM, or extends to other PTMs as well. First, we studied whether the IgMs reacted to different PTMs when present on the same cyclic peptide backbone (C-PTM-P2). Interestingly, 2D5 recognized not only the citrullinated, but also the homocitrullinated version of this peptide. Moreover, the 1G8, also derived from a CCP2-reactive B cell, recognized the citrullinated, homocitrullinated and acetylated version of the peptide, although acetyllysine was recognized to a lesser extent. In contrast, the recognition profile of 1E3 was restricted to the acetyllysine modification (Fig. 2C). Noteworthy, similar results were obtained using a larger panel of PTM peptides and proteins (Fig. 2C, D). As additional control, we generated an IgM mAb against TT, which did not recognize any of the modified peptides or proteins (Fig. 2C, D). As expected, the anti-TT IgM recognized TT in a concentration dependent manner, while the AMPA-IgMs did not bind TT (Figure S4), further strengthening the specificity of the antibodies. The cross-reactivity of the mAbs towards different PTM antigens was confirmed by western blot and a titration ELISA using modified fibrinogen as a model antigen (Fig. 2E and S5A). To further validate the cross-reactivity towards different PTMs, we performed a protein inhibition ELISA for 2D5 that recognizes multiple PTMs as well as the acetyllysine-directed mAb 1E3. As predicted by the titration ELISAs presented above, the recognition of 2D5 could be inhibited by citrullinated and carbamylated proteins, but not by acetylated or unmodified proteins (Figure S5B-C). Likewise, the acetyllysine reactivity of 1E3 could be inhibited by acetylated, but not by citrullinated, carbamylated or unmodified proteins, showing that the recognition of this IgM antibody is confined to acetylated antigens (Figure S5D). Together, these results indicate that AMPA-IgM do not only recognize multiple modified antigens within one specific PTM, but can cross-react towards different PTMs as well, even though they have undergone limited SHM.

### PTM cross-reactivity can be present in germline AMPA-IgM and expands through BCR mutations

**Fig. 3:**
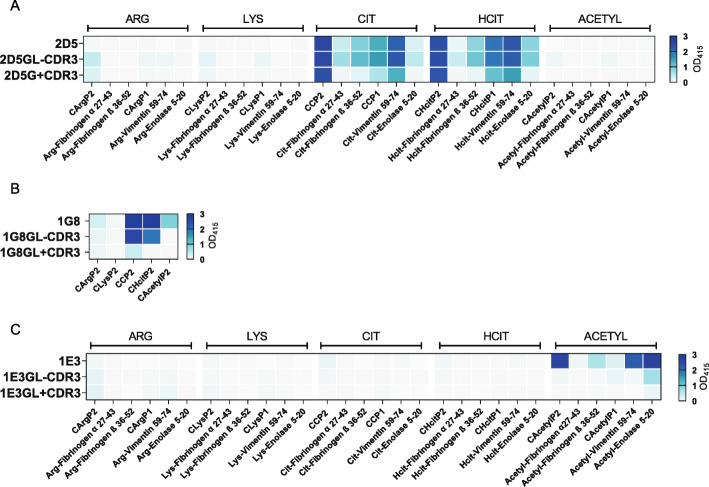
Cross-reactivity of monoclonal AMPA-IgM in germline configuration. Heatmap of a PTM-peptide ELISA with 10 μg/ml mAb and its germline variants (coated 10 μg/ml antigen). **A** 2D5 **B** 1G8 **C** 1E3. All experiments were replicated 2–3 times with similar outcomes. Binding is represented by the optical density at 415 nm

As indicated above, the isolated IgM sequences had undergone limited SHM. Therefore, we next wished to determine whether IgM in germline configuration displays a similar cross-reactivity pattern. The IgM sequences were reverted to their respective germline (GL) sequence (Table S1). Two GL variants of each sequence were generated. First, we generated a variant in which the CDR3-region was unaffected (GL without CDR3) as usually done when generating antibodies in germline configuration. Moreover, we generated a variant in which the CDR3-region, except for junctions and TdT N nucleotides, was reverted into its germline configuration as well (GL with CDR3). The integrity of the antibodies was verified and confirmed with native-PAGE (Figure S6). Interestingly, both germline variants of 2D5 still showed specific reactivity towards citrulline and homocitrulline on multiple backbones. However, the 2D5 GL with CDR3 displayed a slightly reduced reactivity to the lower affine antigens (Fig. 3A). Reversion to GL had a considerable impact on the recognition profile of 1E3 as both germline variants did not show reactivity to most acetylated antigens anymore (Fig. 3C). As purification of the mAb 1G8 GL with CDR3 resulted in aggregation, we identified the reactivity patterns of 1G8 and its GL variants using the HEK cell culture supernatants. As the concentration of the antibody in culture supernatants was relatively low, we did not assay all peptides as performed for 2D5 and 1E3. Nonetheless, 1G8 GL without CDR3 displayed a similar cross-reactivity pattern for CCP2/CHcitP2 as the parental 1G8, whereas reactivity to the acetyllysine was lost. When also the CDR3-region was reverted to germline, reactivity was further diminished, but CCP2 was still recognized (Fig. 3B). Thus, together, these results indicate that AMPA-IgM in germline configuration can recognize various PTM antigens as exemplified by 2D5 and 1G8, but also suggest that SHM increases the number of modified antigens recognized as indicated by the results obtained for all three mAbs.

### Antibody valency is crucial for broad reactivity of AMPA-IgM

**Fig. 4:**
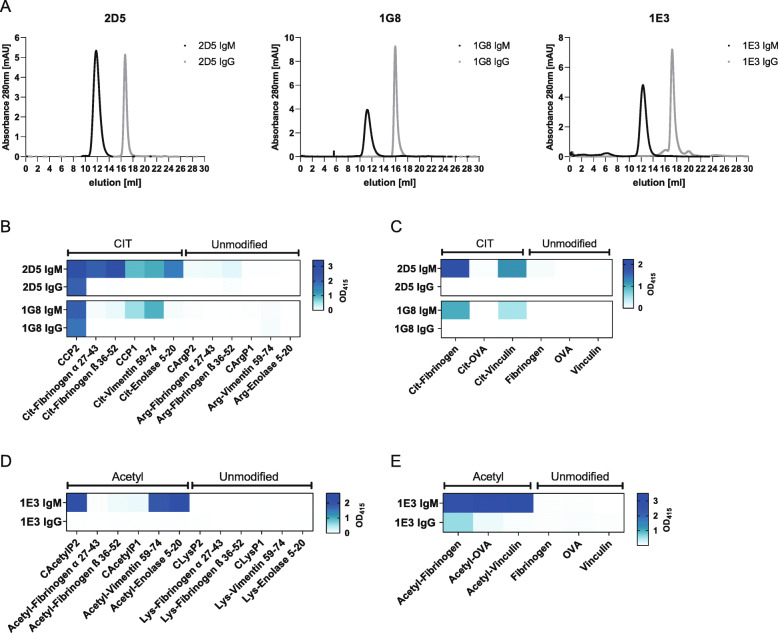
Antibody valency: AMPA-IgM expressed as IgG. **A** Analytical size exclusion chromatography of pentameric IgM and monomeric IgG mAb. 30 µg mAb were loaded on a Superose6Increase 10/30 GL column. **B** ELISA with 2D5 and 1G8 as IgM and as IgG (10 μg/ml) on different citrullinated peptides and arginine-control peptides (coated 10 μg/ml antigen) and **C** citrullinated and unmodified proteins (coated 10 μg/ml antigen). **D** ELISA with 1E3 as IgM and as IgG (10 μg/ml) on different acetylated peptides and lysine-control peptides (coated 10 μg/ml antigen) and **E** acetylated and unmodified proteins (coated 10 μg/ml antigen). All experiments were replicated 2–3 times with similar outcomes. Binding is represented by the optical density at 415 nm

Next, we wished to investigate whether the pentameric structure of the IgM antibodies influences the extent to which different PTM antigens are recognized. Therefore, the pentameric IgM mAbs (valency = 10) were expressed as IgG (valency = 2), leaving the variable domain unaffected. The respective size was confirmed by analytical SEC (Fig. 4A). Recognition of multiple citrullinated antigens, for 2D5 and 1G8, or acetylated antigens, for 1E3, was determined for both the IgM and IgG antibodies. The experiments were performed with identical protein concentrations of IgM and IgG mAbs, ensuring that an equal amount of binding arms is present. Interestingly, recognition of all citrullinated antigens, except CCP2 antigen, was lost when 2D5 and 1G8 were expressed as IgG (Fig. 4B, C). Likewise, 1E3 expressed as IgG only recognized acetylated fibrinogen, whereas 1E3 as IgM could recognize all acetylated proteins and 3 out of 6 acetylated peptides that were tested (Fig. 4D, E). In contrast, when variable regions obtained from IgG expressing B cells were expressed as IgG as control, all mAbs recognized multiple PTM antigens [10]. These results indicate that antibody valency impacts the recognition profile and binding capacity of AMPA-IgM to multiple PTM antigens and that germline variants of IgG might test negative when analysed in the absence of appropriate valency.

### AMPA-IgM can activate complement more efficiently than AMPA-IgG

**Fig. 5:**
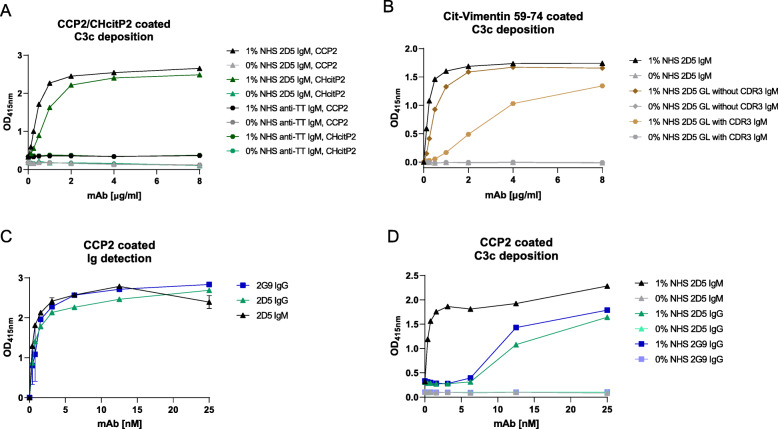
Complement activation of AMPA IgM. **A** Complement C3c deposition ELISA of the mAb TT-IgM and 2D5-IgM on a CCP2 (1 μg/ml) or CHcitP2 (1 μg/ml) coated plate. **B** C3c desposition ELISA with 2D5-IgM and and 2D5 GL with and without CDR3 mAb bound to Cit-vimentin 59-74 (coated 10 μg/ml antigen). **C** CCP2 titration ELISA of 2D5-IgM, 2D5-IgG and 2G9-IgG mAbs using equal molarities (coated 1 μg/ml CCP2). **D** C3c deposition ELISA with 2D5-IgM, 2D5-IgG and 2G9-IgG mAbs using equal molarities (coated 1 μg/ml CCP2). All experiments were replicated 2–3 times with similar outcomes

As IgM is typically the first isotype to appear during an antibody response, we wished to determine whether AMPA-IgM could, potentially, initiate inflammation. Although, it is known that IgM can activate the complement system efficiently, it is unclear if or how efficiently AMPA-IgM can activate complement, as compared to AMPA-IgG. Therefore, we investigated the potential of AMPA-IgM to activate the classical complement pathway as measured by C3c deposition. Indeed, binding of 2D5, but not of the anti-TT-IgM as control, to citrulline- and homocitrulline-containing antigens led to deposition of C3c in the presence of normal human serum (NHS) (Fig. 5A). Complement activation was not only observed using CCP2 as antigenic target for AMPA-IgM, but also other peptides such as citrullinated vimentin (Fig. 5B). Further, C3c deposition was observed for the AMPA-IgM 2D5 germline variants as well, not only indicating that also AMPA in germline configuration can activate complement, but also that recognition multiple antigens/PTMs can trigger such activation (Fig. 5B). To demonstrate the relative impact of AMPA-IgM on complement activation in comparison to AMPA-IgG, we next evaluated the capacities of the 2D5 in both the IgM and IgG variant to activate complement. The two isotypes were compared on a molar basis. The IgM antibody was approximately 30-fold more efficient to mediate C3c deposition (Fig. 5D) despite the high binding of both isotypes to the antigen (Fig. 5C). To validate these results further, we next wished to compare the ability to activate complement to another IgG (2G9-IgG) that was not derived from an IgM AMPA-sequence but has been isolated from a ACPA-IgG-expressing B cell [10]. The antigen binding of the 2D5-IgM and 2G9-IgG were similar (Fig. 5C), but again, AMPA-IgM was more efficient in complement activation as 2D5-IgM mediated the same C3c deposition in an approximately 15-fold lower concentration as compared to 2G9-IgG (Fig. 5D). These data indicate that AMPA-IgM, already in their germline configuration, can play a prominent role in complement activation and, potentially, subsequent inflammation in RA.

## Discussion

This study shows that AMPA-IgM not only recognize multiple antigens expressing the same modification, but also that it can be cross-reactive towards different PTMs as well. Intriguingly, this is also the case for germline AMPA-IgM, exemplarily shown for the 2D5 IgM, which depicts a limited amount of mutations and could thus be reverted to its germline configuration. These results indicate that the initial breach of B cell tolerance towards PTM antigens could be induced by different antigens and modifications, leading to a cross-reactive AMPA response. Likewise, since SHM, such as observed in AMPA-IgM, is only introduced after BCR-mediated B cell activation, it is probable that the initial recognition of PTM antigens is sufficient to trigger naïve B cells and thus induce autoimmunity.

Our data show that pentameric IgM AMPA can recognize multiple modified antigens, but that this recognition is largely lost when antibodies are produced as monomeric IgG. Although the latter might not be unexpected as IgM displays a 5 times higher valency as compared to IgG, the data indicate that generation of pentameric IgM was crucial to show that already a limited number of somatic mutations is sufficient to allow for a broad PTM-recognition profile. These findings would have been missed in case the parental sequences would have been expressed as IgG only as these did not recognize multiple PTM-epitopes. Our data also indicate that IgM antibodies in germline configuration, although still reactive to PTMs, are less polyreactive towards several modified antigens. These data are in line with observations indicating that SHM observed in ACPA-IgG-molecules allow binding to different citrullinated antigens and/or polyreactivity to multiple citrullinated epitopes [18, 22]. The notion that with the accumulation of Ig somatic mutations, ‘breadth’ of an antibody response increases is reminiscent to recent observations made by analysing the evolution of the antibody response against COVID-19 in individuals that recovered from infection [31]. In this study, it was shown that monoclonal antibodies to SARS-CoV-2 isolated long after infection were able to recognize more viral variants carrying receptor-binding domain (RBD) mutations at the antibody-binding site as compared to their ‘ancestral’ counterparts isolated shortly after infection. Thus, also in this case, the ‘breadth’, i.e. the ability to recognize more related epitopes, of the antibody response was increased upon increased SHM.

Although our data indicate that recognition of PTM antigens by naïve B cells can induce AMPA-B cell responses, they do not exclude the possibilty that such responses can also arise as a consequence of SHM. For example, in the autoimmune disease pemphigus vulgaris, autoantibodies against desmoglein (DSG) protein 3 have been reported to lose reactivity to DSG3 when reverted to germline [19]. Similar observations have been made for anti-DNA IgG antibodies in systemic lupus erythematosus and GM-CSF IgG autoantibodies in pulmonary alveolar proteinosis [20, 21, 32]. These observations are in line with our observations on the IgM monoclonal 1E3 for which the reactivity to acetyllysine was undetectable when reverted to germline. While this latter observation might also be explained by a possible deviation from the original germline sequence, it is in line with the notion that PTM-reactivity can be introduced after SHM. Interestingly, the 1E3 is, to the best of our knowledge, the first monclonal antibody which only recognizes acetylated antigens and not citrullinated or carbamylated antigens that is isolated from an RA patient. This is possibly explained best by the fact that the 1E3 was derived from a B cell isolated with a acetylated peptide and indicates that anti-acetyllysine reactivity is not necessarily derived from ACPA expressing B cells. It will be relevant to analyse to which extent the introduction of (further) PTM-reactivity relies on SHM to unrelated antigens, such as present in the microbiota.

In RA, there has been a major focus on AMPA-IgG as this is the predominant isotype found in sera of RA patients. However, IgM ACPA are also present in a substantial number of CCP+ RA-patients, especially in synovial fluid in which elevated levels of IgM ACPA have been described [33]. We now show that AMPA-IgM are also potent inducers of complement activation, indicating that the contribution of AMPA-IgM in the pathogenesis of RA might be underestimated. Indeed, joint inflammation is strongly associated with complement activation in RA patients as increased levels of complement components are present in synovial fluid and complement deposition can be demonstrated in synovial tissue [34]. If or how ACPA IgM contributes to synovial inflammation is unknown, but our data indicate that they might be able to do so as complement activation is 15–30 times more potent compared to ACPA-IgG.

A limitation of the study is that we succeeded in producing only a limited number of AMPA-IgM. This is partly explained by our observation that the number of AMPA-IgM expressing B cells in the circulation of RA patients is low. Additionally, production of antigen-specific IgMs is challenging, as also evidenced by a recent study in which 46 IgM sequences from COVID-19-specific B cells were tested as IgG antibodies because of the difficulty in producing IgM pentamers [35]. Clearly, the limited number of unique IgM molecules produced limits the generalizability of the data presented. Nonetheless, they do reveal important observations as they show that IgM ACPA can be cross-reactive towards different citrullinated antigens as well as different PTMs. Likewise, the data indicate that AMPA-reactivity can be encoded within the naïve B cell receptor repertoire as also the IgM molecules reverted to germline did show reactivity to PTM antigens.

## Conclusions

Collectively, our study shows that the breach of B cell tolerance against PTM antigens could already occur before the B cells have undergone (extensive) SHM and class switch recombination. Moreover, we show a broad PTM antigen recognition profile of AMPA-IgM, indicating that a variety of antigens could be involved in the initial B cell activation and/or the perpetuation of the IgM response in the inflamed joint. Likewise, our results indicate that AMPA-IgM could be involved in synovial inflammation by facilitating complement activation.

## Supplementary Information

**Additional file 1.** Supplementary material and methods.

**Additional file 2.** Figure S1.

**Additional file 3.** Figure S2.

**Additional file 4.** Figure S3.

**Additional file 5.** Figure S4.

**Additional file 6.** Figure S5.

**Additional file 7.** Figure S6.

## Data Availability

All data generated or analysed during this study are included in this published article and its supplementary information files or are available from the corresponding author on reasonable request.

## Abbreviations

RA: Rheumatoid arthritis
ACPA: Anti-citrullinated protein antibody
PTM: Post-translational modification
aCarPA: Anti-carbamylated protein antibody
AAPA: Anti-acetylated protein antibody
AMPA: Anti-modified protein antibody
BCR: B cell receptor
SHM: Somatic hypermutation
HC: Heavy chain
LC: Light chain
V: Variable
D: Diversity
J: Joining
TdT: Deoxynucleotidyl transferase
TT: Tetanus toxoid
NHS: Normal human serum
CCP: Cyclic citrullinated peptide
GL: Germline
SEC: Size exclusion chromatography
DSG: Desmoglein

## References

[1] Schellekens GA, de Jong BA, van den Hoogen FH, van de Putte LB, van Venrooij WJ (1998). Citrulline is an essential constituent of antigenic determinants recognized by rheumatoid arthritis-specific autoantibodies. J Clin Invest.

[2] Ioan-Facsinay A, El-Bannoudi H, Scherer HU, Van Der Woude D, Menard HA, Lora M (2011). Anti-cyclic citrullinated peptide antibodies are a collection of anti-citrullinated protein antibodies and contain overlapping and non-overlapping reactivities. Ann Rheum Dis.

[3] Pratesi F, Panza F, Paolini I, Petrelli F, Puxeddu I, Casigliani-Rabl S, Ancillotti D, Alcaro C, Rovero P, Migliorini P (2015). Fingerprinting of anti-citrullinated protein antibodies (ACPA): specificity, isotypes and subclasses. Lupus..

[4] Ge C, Xu B, Liang B, Lonnblom E, Lundstrom SL, Zubarev RA (2019). Structural basis of cross-reactivity of anti-citrullinated protein antibodies. Arthritis Rheum.

[5] Shi J, Knevel R, Suwannalai P, van der Linden MP, Janssen GMC, van Veelen PA, Levarht NEW, van der Helm-van Mil AHM, Cerami A, Huizinga TWJ, Toes REM, Trouw LA (2011). Autoantibodies recognizing carbamylated proteins are present in sera of patients with rheumatoid arthritis and predict joint damage. Proc Natl Acad Sci.

[6] Juarez M, Bang H, Hammar F, Reimer U, Dyke B, Sahbudin I, Buckley CD, Fisher B, Filer A, Raza K (2016). Identification of novel antiacetylated vimentin antibodies in patients with early inflammatory arthritis. Ann Rheum Dis.

[7] Jiang X, Trouw LA, Van Wesemael TJ, Shi J, Bengtsson C, Källberg H (2014). Anti-CarP antibodies in two large cohorts of patients with rheumatoid arthritis and their relationship to genetic risk factors, cigarette smoking and other autoantibodies. Ann Rheum Dis.

[8] Shi J, Willemze A, Janssen GMC, Van Veelen PA, Drijfhout JW, Cerami A (2013). Recognition of citrullinated and carbamylated proteins by human antibodies: specificity, cross-reactivity and the ‘AMC-Senshu’ method. Ann Rheum Dis.

[9] Reed E, Jiang X, Kharlamova N, Ytterberg AJ, Catrina AI, Israelsson L, Mathsson-Alm L, Hansson M, Alfredsson L, Rönnelid J, Lundberg K (2016). Antibodies to carbamylated alpha-enolase epitopes in rheumatoid arthritis also bind citrullinated epitopes and are largely indistinct from anti-citrullinated protein antibodies. Arthritis Res Ther.

[10] Kissel T, Reijm S, Slot L, Cavallari M, Wortel C, Vergroesen R, et al. Antibodies and B cells recognising citrullinated proteins display a broad cross-reactivity towards other post-translational modifications. Ann Rheum Dis. 2020; annrheumdis-201. 10.1136/annrheumdis-2019-216499 32041746

[11] Steen J, Forsstrom B, Sahlstrom P, Odowd V, Israelsson L, Krishnamurthy A (2019). Recognition of amino acid motifs, rather than specific proteins, by human plasma cell-derived monoclonal antibodies to posttranslationally modified proteins in rheumatoid arthritis. Arthritis Rheum.

[12] Lloyd KA, Wigerblad G, Sahlström P, Garimella MG, Chemin K, Steen J, et al. Differential ACPA binding to nuclear antigens reveals a PAD-independent pathway and a distinct subset of acetylation cross-reactive autoantibodies in rheumatoid arthritis. Front Immunol. 2019;9. 10.3389/fimmu.2018.03033. 10.3389/fimmu.2018.03033 PMC6328449 30662440

[13] Kampstra ASB, Dekkers JS, Volkov M, Dorjée AL, Hafkenscheid L, Kempers AC, van Delft M, Kissel T, Reijm S, Janssen GMC, van Veelen PA, Bang H, Huizinga TWJ, Trouw LA, van der Woude D, Toes REM (2019). Different classes of anti-modified protein antibodies are induced on exposure to antigens expressing only one type of modification. Ann Rheum Dis.

[14] Suwannalai P, Willemze A, van Toorn L, Stoeken-Rijsbergen G, Levarht N, Drijfhout JW, J Huizinga TW, M Toes RE, Trouw LA (2011). The fine specificity of IgM anti-citrullinated protein antibodies (ACPA) is different from that of IgG ACPA. Arthritis Res Ther.

[15] De Moel EC, Derksen VFAM, Stoeken G, Trouw LA, Bang H, Goekoop RJ, et al. Baseline autoantibody profile in rheumatoid arthritis is associated with early treatment response but not long-term outcomes. Arthritis Res Ther. 2018;20(1). 10.1186/s13075-018-1520-4 PMC5828136 29482627

[16] Ioan-Facsinay A, Willemze A, Robinson DB, Peschken CA, Markland J, Van Der Woude D (2008). Marked differences in fine specificity and isotype usage of the anti-citrullinated protein antibody in health and disease. Arthritis Rheum.

[17] Vergroesen RD, Slot LM, Hafkenscheid L, Koning MT, Van Der Voort EIH, Grooff CA, et al. B-cell receptor sequencing of anti-citrullinated protein antibody (ACPA) IgG-expressing B cells indicates a selective advantage for the introduction of N -glycosylation sites during somatic hypermutation. Ann Rheum Dis. 2017; annrheumdis-201. 10.1136/annrheumdis-2017-212052 28835463

[18] Elliott SE, Kongpachith S, Lingampalli N, Adamska JZ, Cannon BJ, Mao R, Blum LK, Robinson WH (2018). Affinity maturation drives epitope spreading and generation of proinflammatory anti–citrullinated protein antibodies in rheumatoid arthritis. Arthritis Rheum.

[19] Di Zenzo G, Di Lullo G, Corti D, Calabresi V, Sinistro A, Vanzetta F (2012). Pemphigus autoantibodies generated through somatic mutations target the desmoglein-3 cis-interface. J Clin Investig.

[20] Wellmann U, Letz M, Herrmann M, Angermuller S, Kalden JR, Winkler TH (2005). The evolution of human anti-double-stranded DNA autoantibodies. Proc Natl Acad Sci U S A.

[21] Piccoli L, Campo I, Fregni CS, Rodriguez BMF, Minola A, Sallusto F, Luisetti M, Corti D, Lanzavecchia A (2015). Neutralization and clearance of GM-CSF by autoantibodies in pulmonary alveolar proteinosis. Nat Commun.

[22] Kongpachith S, Lingampalli N, Ju CH, Blum LK, Lu DR, Elliott SE, Mao R, Robinson WH (2019). Affinity maturation of the anti-citrullinated protein antibody paratope drives epitope spreading and polyreactivity in rheumatoid arthritis. Arthritis Rheum.

[23] Ozawa T, Ouhara K, Tsuda R, Munenaga S, Kurihara H, Kohno H, Hamana H, Kobayashi E, Taki H, Tobe K, Sugiyama E, Muraguchi A, Kishi H (2020). Physiological target, molecular evolution and pathogenic functions of a monoclonal ACPA obtained from an RA patient. Arthritis Rheum.

[24] Trouw LA, Haisma EM, Levarht EWN, Van Der Woude D, Ioan-Facsinay A, Daha MR (2009). Anti-cyclic citrullinated peptide antibodies from rheumatoid arthritis patients activate complement via both the classical and alternative pathways. Arthritis Rheum.

[25] Figueiredo CP, Bang H, Cobra JF, Englbrecht M, Hueber AJ, Haschka J, Manger B, Kleyer A, Reiser M, Finzel S, Tony HP, Kleinert S, Wendler J, Schuch F, Ronneberger M, Feuchtenberger M, Fleck M, Manger K, Ochs W, Schmitt-Haendle M, Lorenz HM, Nuesslein H, Alten R, Henes J, Krueger K, Rech J, Schett G (2017). Antimodified protein antibody response pattern influences the risk for disease relapse in patients with rheumatoid arthritis tapering disease modifying antirheumatic drugs. Ann Rheum Dis.

[26] Lighaam LC, Vermeulen E, Den Bleker T, Meijlink KJ, Aalberse RC, Barnes E (2014). Phenotypic differences between IgG4+ and IgG1+ B cells point to distinct regulation of the IgG4 response. J Allergy Clin Immunol.

[27] Huijbers MG, Vergoossen DL, Fillié-Grijpma YE, Van Es IE, Koning MT, Slot LM (2019). MuSK myasthenia gravis monoclonal antibodies. Neurol Neuroimmunol Neuroinflamm.

[28] Koning MT, Kiełbasa SM, Boersma V, Buermans HPJ, Van Der Zeeuw SAJ, Van Bergen CAM (2017). ARTISAN PCR: rapid identification of full-length immunoglobulin rearrangements without primer binding bias. Br J Haematol.

[29] Brochet X, Lefranc MP, Giudicelli V (2008). IMGT/V-QUEST: the highly customized and integrated system for IG and TR standardized V-J and V-D-J sequence analysis. Nucleic Acids Res.

[30] Kristyanto H, Blomberg NJ, Slot LM, Van Der Voort EIH, Kerkman PF, Bakker A (2020). Persistently activated, proliferative memory autoreactive B cells promote inflammation in rheumatoid arthritis. Sci Transl Med.

[31] Gaebler C, Wang Z, Lorenzi JCC, Muecksch F, Finkin S, Tokuyama M, Cho A, Jankovic M, Schaefer-Babajew D, Oliveira TY, Cipolla M, Viant C, Barnes CO, Bram Y, Breton G, Hägglöf T, Mendoza P, Hurley A, Turroja M, Gordon K, Millard KG, Ramos V, Schmidt F, Weisblum Y, Jha D, Tankelevich M, Martinez-Delgado G, Yee J, Patel R, Dizon J, Unson-O’Brien C, Shimeliovich I, Robbiani DF, Zhao Z, Gazumyan A, Schwartz RE, Hatziioannou T, Bjorkman PJ, Mehandru S, Bieniasz PD, Caskey M, Nussenzweig MC (2021). Evolution of antibody immunity to SARS-CoV-2. Nature..

[32] Mietzner B, Tsuiji M, Scheid J, Velinzon K, Tiller T, Abraham K, Gonzalez JB, Pascual V, Stichweh D, Wardemann H, Nussenzweig MC (2008). Autoreactive IgG memory antibodies in patients with systemic lupus erythematosus arise from nonreactive and polyreactive precursors. Proc Natl Acad Sci.

[33] Willemze A, Shi J, Mulder M, Stoeken-Rijsbergen G, Drijfhout JW, Huizinga TWJ, Trouw LA, Toes REM (2013). The concentration of anticitrullinated protein antibodies in serum and synovial fluid in relation to total immunoglobulin concentrations. Ann Rheum Dis.

[34] Sturfelt G, Truedsson L (2012). Complement in the immunopathogenesis of rheumatic disease. Nat Rev Rheumatol.

[35] Wang Z, Lorenzi JCC, Muecksch F, Finkin S, Viant C, Gaebler C (2021). Enhanced SARS-CoV-2 neutralization by dimeric IgA. Sci Transl Med.

