# Supplementary material for: Cross-reactivity of IgM anti-modified protein antibodies in rheumatoid arthritis despite limited mutational load
Source: Arthritis Res Ther. 2021 Sep 3;23:230. doi: 10.1186/s13075-021-02609-5 (PMC8413699; doi:10.1186/s13075-021-02609-5)
Supplement: Supplementary file 1 — Additional file 1. Supplementary material and methods. [file 13075_2021_2609_MOESM1_ESM.docx]

**Supplementary material and methods**

**Protein modification and peptide synthesis**

Vinculin, Fibrinogen, and Ovalbumin (OVA, Sigma-Aldrich) protein modification (citrullination, carbamylation and acetylation) as well as FCS carbamylation was performed as previously described.(1, 2) Four linear N-terminal biotinylated peptides (Fibrinogen alpha 27-42, Fibrinogen beta 36-52, Vimentin 59-74 and Enolase 5-20) as well as 2 cyclic N-terminal biotinylated peptides (C(C/Hcit/Acetyl)P1 and C(C/Hcit/Acetyl)P2), were synthesized in 5 different modifications including the altered amino acid residue at the same positions within the peptide sequence (citrulline, arginine, homocitrulline, acetyl-lysine, lysine). Sequences are given in Table S2. Peptide synthesis was performed and the integrity of the synthesized peptides was verified by HPLC and mass spectrometry data as previously published (3).

**Antigen labelling**

The CCP2 and CargP2 tetramers were made as previously described.(4) The same method was used to produce Ac-Vimentin (HC55, Orgentec) and Lys-Vimentin (HC56, Orgentec) tetramers for capturing of AAPA expressing B cells. To identify tetanus-specific B cells, Tetanus Toxoid (Statens Serum Institute) was labelled with APC or PE using AnaTag labelling kit, according to the manufacturer’s instruction (AnaSpec). Optimal concentrations of labelled tetramers and of labelled Tetanus Toxoid (TT) were obtained by titration on HEK^ACPA-TM^ and wild type HEK 293T cells or on immortalized TT-specific B cells, respectively.

**Purification of polyclonal and monoclonal AMPA-IgM**

In order to isolated the polyclonal and monoclonal antibodies, 1mg biotinylated CCP2 or biotinylated CAcetylP2 were coupled to a HiTrap Streptavidin HP column (Cytivia). The antibodies were purified using a tandem purification method. The transfection supernatant was applied to the CCP2 or CAcetylP2 column and eluted with glycine-HCL buffer of pH 3.5. Buffer exchange to PBS was performed immediately after CCP2/CAcetylP2 purification using the HiPrep^TM^ 26/10 Desalting column (Cytivia) which was connected downstream. All purifications were performed using an AKTA pure protein purification system.

**Size** **exclusion chromatography (SEC)**

To identify the size, purified monoclonal antibodies (30μg) were loaded on a Superose6Increase 10/30 GL column on an AKTA pure protein system and eluted with PBS for 1,5 column volumes. UV was monitored at 280nm.

**Native gel**

1-3 µg of mononoclonal IgM were diluted in TRIS glycine native sample buffer (Novex) and loaded on a 7% TRIS-acetate gel (Novex NuPAGE) in native running buffer (Novex) together with NativeMark unstained protein standard (Invitrogen). The native gel was stained with SimplyBlue SafeStain (Invitrogen) for 1h and destained in distilled water.

**Supplementary Tables and Figures**

**Figure S1.** ACPA IgM ELISA mixing RF+/- and ACPA+/- plasma of RA patients. The CCP2-IgG+ plasmas mixed with RF-IgM+ plasma are shown in orange dots.

**Figure S2.** A) Gating strategy of the single cell sort. The HC55+ antigen sort is shown here as example. B) Backgating of the 1E3 sorted B cell. The same gates are used as shown in the gating strategy.

**Figure S3. A)** ELISA with the patient B cell culture supernatants and the respective pentameric IgM mAb (10μg/ml) on the antigen used for B cell isolation. The respective arginine or lysine containing control peptide is included. Binding is represented by the optical density at 415nm. **B)** Analytical size exclusion chromotography (SEC). 20-40µg mAb IgM were loaded on a Superose6Increase 10/30 GL column. **C)** Native 7% TRIS acetate gel using 3 µg mAbs.

**Figure S4.** TT titration ELISA with 2D5, 1G8, 1E3 and anti-TT-IgM.

**Figure S5.** **A)** PTM-fibrinogen titration ELISA with 2D5, 1G8 and 1E3 mAb (coated 10μg/ml). **B-C)** Cross-inhibition ELISA with PTM-Fibrinogen for 2D5 mAb (coated 10μg/ml). **D)** Cross-inhibition ELISA with PTM-Fibrinogen for 1E3 mAb (coated 10μg/ml).

**Figure S6.** Native gel of 1-3 μg 2D5 and 1E3 IgM monoclonal antibodies and the respective germline variants.

**Table S1.** Germline reverted amino acids (aa) of the heavy chain (HC) and light chain (LC) of 2D5, 1G8 and 1E3.

| **Antibody chain** | **Reverted aa** | **# reverted aa** |
| --- | --- | --- |
| 2D5 HC germline – CDR3 | T>K (FR1), T>S (CDR1) | 2 |
| 2D5 HC germline + CDR3 | T>K (FR1), T>S (CDR1), A>G (CDR3) | 3 |
| 2D5 LC germline – CDR3 | E>K (FR1), K>R (FR3), F>L (FR3) | 3 |
| 2D5 LC germline + CDR3 | E>K (FR1), K>R (FR3), F>L (FR3), N>S (CDR3) | 4 |
| 1G8 HC germline | N>Y (CDR1), I>M (CDR1), D>N (CDR2), R>S (FR3), S>A) FR3) | 5 |
| 1G8 LC germline – CDR3 | A>S (FR1), V>L (FR1), L>Q (FR2), T>S (CDR2) | 4 |
| 1G8 LC germline + CDR3 | A>S (FR1), V>L (FR1), L>Q (FR2), T>S (CDR2), D>G (CDR3) | 5 |
| 1E3 HC germline – CDR3 | S>T (FR1), D>G (CDR1), A>G (FR2), H>Y (CDR2), S>N (CDR2), M>V (FR3), N>K (FR3), N>S (FR3), T>V (FR3) | 9 |
| 1E3 HC germline + CDR3 | S>T (FR1), D>G (CDR1), A>G (FR2), H>Y (CDR2), S>N (CDR2), M>V (FR3), N>K (FR3), N>S (FR3), T>V (FR3), T>R (CDR3), G>D (CDR3), Y>G (CDR3), P>G (CDR3) | 13 |
| 1E3 LC germline – CDR3 | S>P (FR1), K>N (CDR1), E>D (FR2), A>G (CDR2) | 4 |
| 1E3 LC germline + CDR3 | S>P (FR1), K>N (CDR1), E>D (FR2), A>G (CDR2), N>T (CDR3) | 5 |

**Table S2.** PTM-peptide sequences.

| **Peptide** | **aa-sequence** | **number of epitopes** |
| --- | --- | --- |
| HC55 vimentin | Ttds-O-Ttds-GRVYAT-(ac)K-SSAVR | 1 |
| HC56 vimentin | Ttds-O-Ttds-GRVYAT-K-SSAVR |  |
| CCP1 (cyclic) | HQCHQESTXGRSRGRCGRSGSZO | 1 |
| CCP2 (cyclic) | Patent EP2071335 | unknown |
| fibrinogen α 27-43 (linear) | FLAEGGGVXGPRVVERHZO | 1 |
| fibrinogen β 36-52 (linear) (6)(5)[5]^6^ | NEEGFFSAXGHRPLDKKZO | 1 |
| vimentin 59-74 (linear) | VYATXSSAVXLXSSVPZO | 3 |
| enolase 5-20 (linear) | KIHAXEIFDSXGNPTVZO | 2 |

X = arginine, lysine, citrulline, homocitrulline, acetyllysine.

Z = 6-aminohexanoic acid

O = lys (biotine)-amide

Ttds-linker = 1,13-diamino-4,7,10-trioxatridecane succinimic acid linker

**References**

1. Kampstra ASB, Dekkers JS, Volkov M, Dorjée AL, Hafkenscheid L, Kempers AC, et al. Different classes of anti-modified protein antibodies are induced on exposure to antigens expressing only one type of modification. Annals of the Rheumatic Diseases. 2019;78(7):908-16.

2. Shi J, Knevel R, Suwannalai P, van der Linden MP, Janssen GMC, van Veelen PA, et al. Autoantibodies recognizing carbamylated proteins are present in sera of patients with rheumatoid arthritis and predict joint damage. Proceedings of the National Academy of Sciences. 2011;108(42):17372-7.

3. Kissel T, Reijm S, Slot L, Cavallari M, Wortel C, Vergroesen R, et al. Antibodies and B cells recognising citrullinated proteins display a broad cross-reactivity towards other post-translational modifications. Annals of the Rheumatic Diseases. 2020:annrheumdis-201.

4. Kerkman PF, Fabre E, van der Voort EIH, Zaldumbide A, Rombouts Y, Rispens T, et al. Identification and characterisation of citrullinated antigen-specific B cells in peripheral blood of patients with rheumatoid arthritis. Annals of the Rheumatic Diseases. 2016;75(6):1170-6.

5. De Moel EC, Derksen VFAM, Stoeken G, Trouw LA, Bang H, Goekoop RJ, et al. Baseline autoantibody profile in rheumatoid arthritis is associated with early treatment response but not long-term outcomes. Arthritis Research & Therapy. 2018;20(1).

6. van Beers JJ, Willemze A, Jansen JJ, Engbers GH, Salden M, Raats J, et al. ACPA fine-specificity profiles in early rheumatoid arthritis patients do not correlate with clinical features at baseline or with disease progression. Arthritis Res Ther. 2013;15(5):R140.
