# Supplementary figures and images for: Cross-reactivity of IgM anti-modified protein antibodies in rheumatoid arthritis despite limited mutational load
Source: Arthritis Res Ther. 2021 Sep 3;23:230. doi: 10.1186/s13075-021-02609-5 (PMC8413699; doi:10.1186/s13075-021-02609-5)

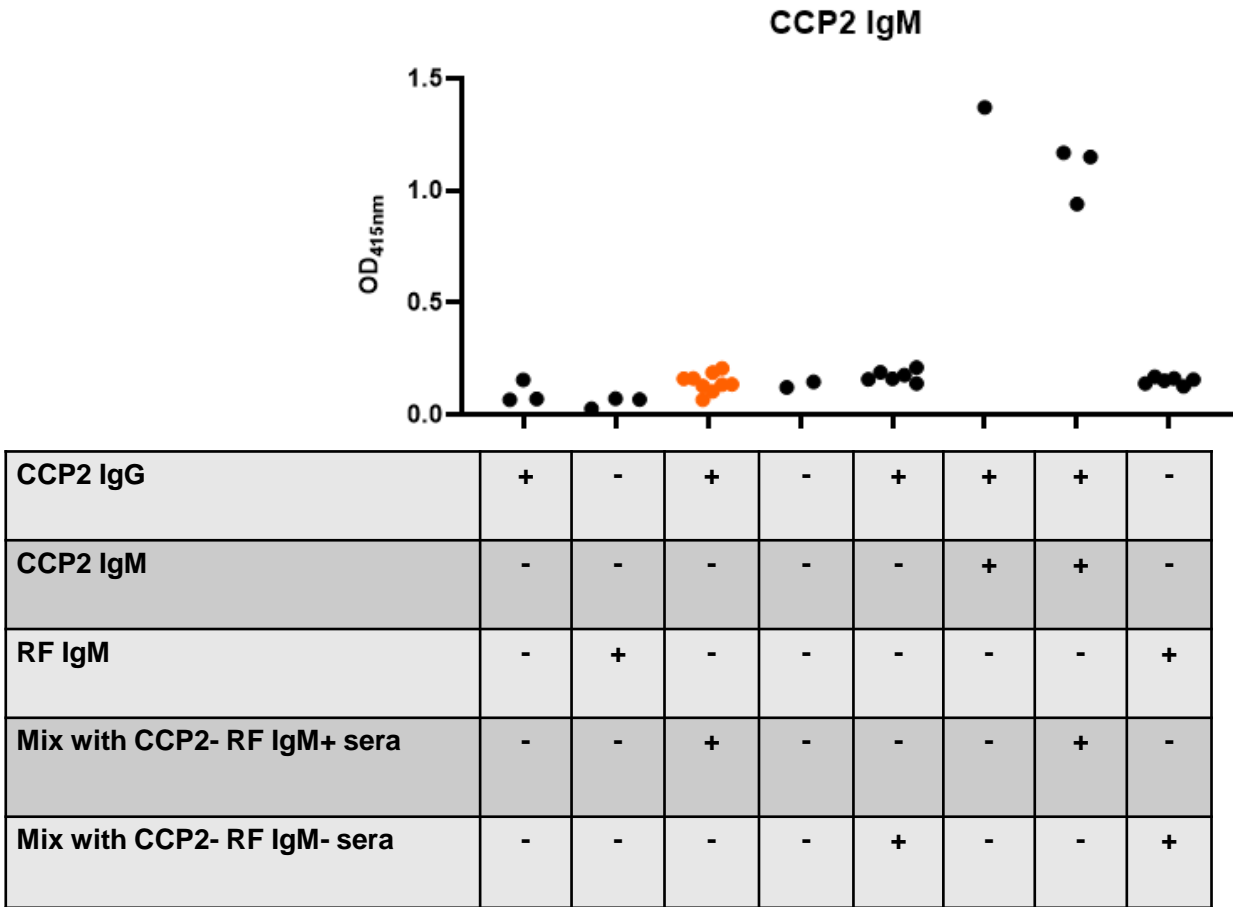

|                             |   |   |   |   |   |   |   |   |
|-----------------------------|---|---|---|---|---|---|---|---|
| CCP2 IgG                    | + | - | + | - | + | + | + | - |
| CCP2 IgM                    | - | - | - | - | - | + | + | - |
| RF IgM                      | - | + | - | - | - | - | - | + |
| Mix with CCP2- RF IgM+ sera | - | - | + | - | - | - | + | - |
| Mix with CCP2- RF IgM- sera | - | - | - | - | + | - | - | + |

Supplement: Supplementary file 2 — Additional file 2. Figure S1. [file 13075_2021_2609_MOESM2_ESM.pdf]

A

## Gating strategy

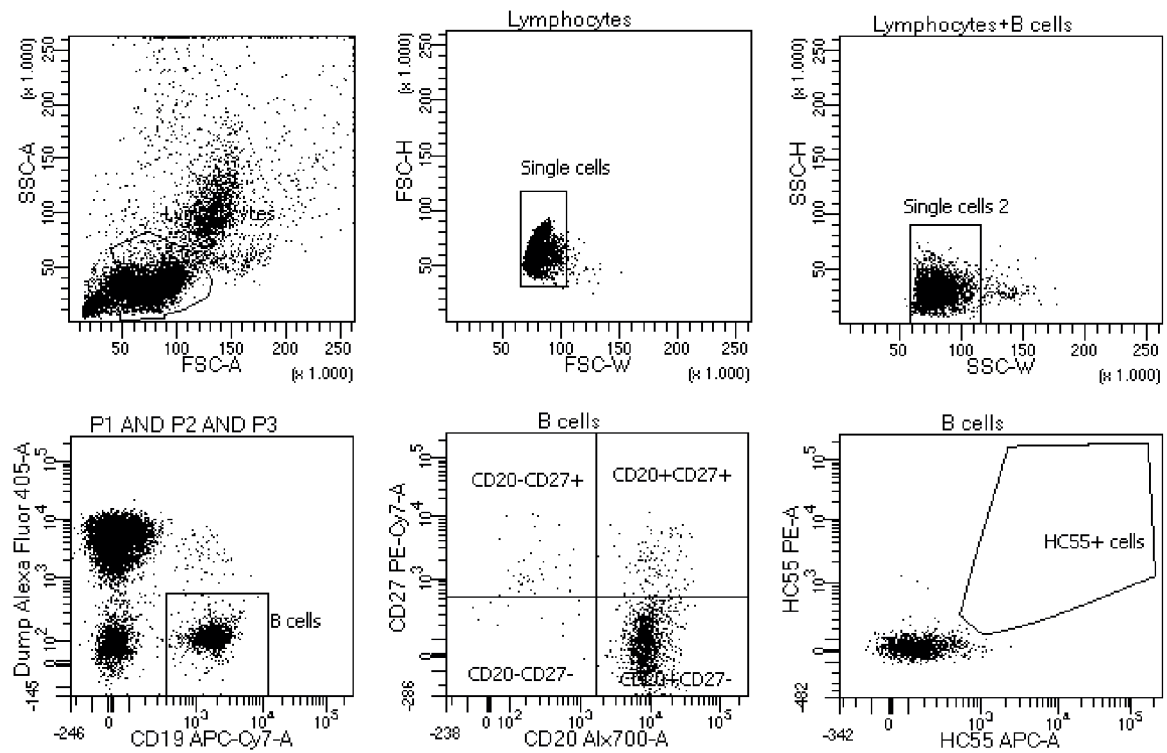

B

## Backgating 1E3

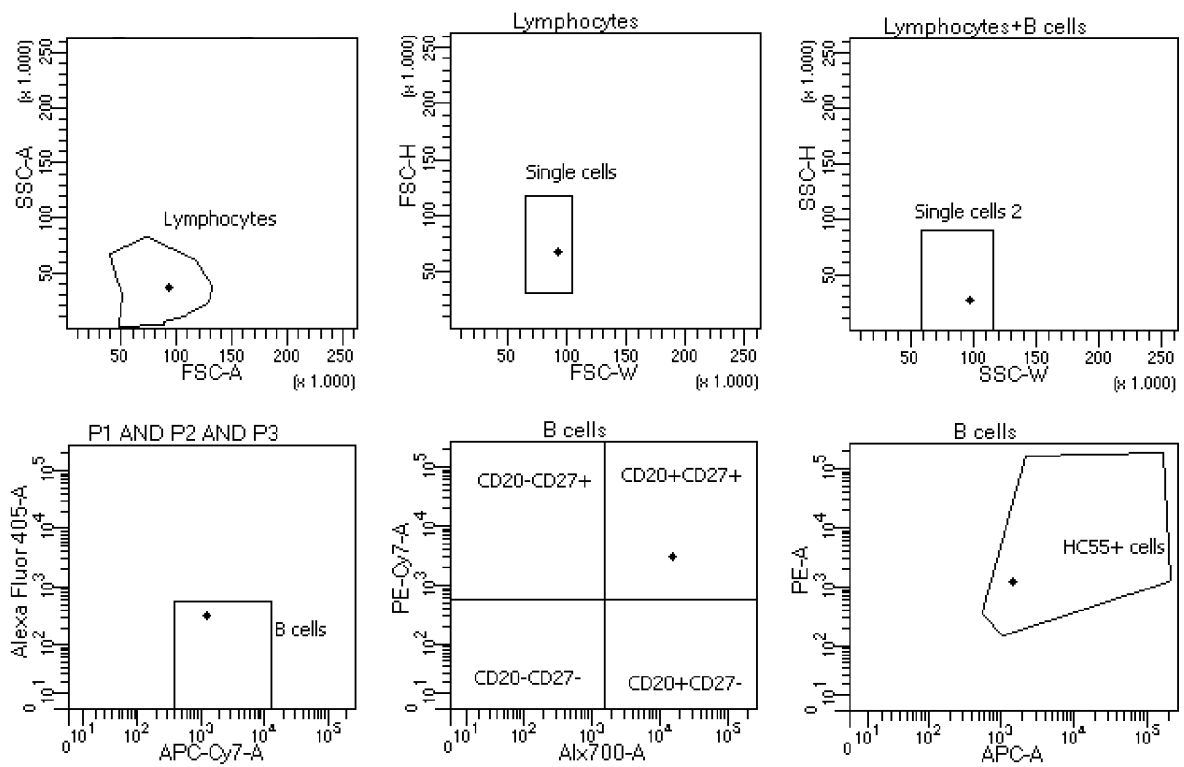

Supplement: Supplementary file 3 — Additional file 3. Figure S2. [file 13075_2021_2609_MOESM3_ESM.pdf]

A

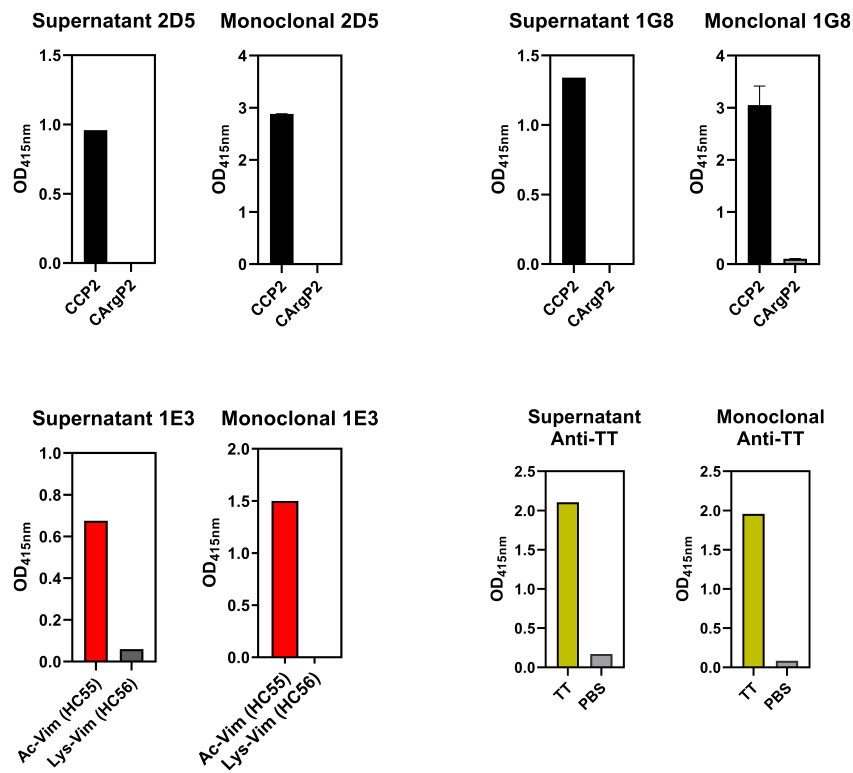

B

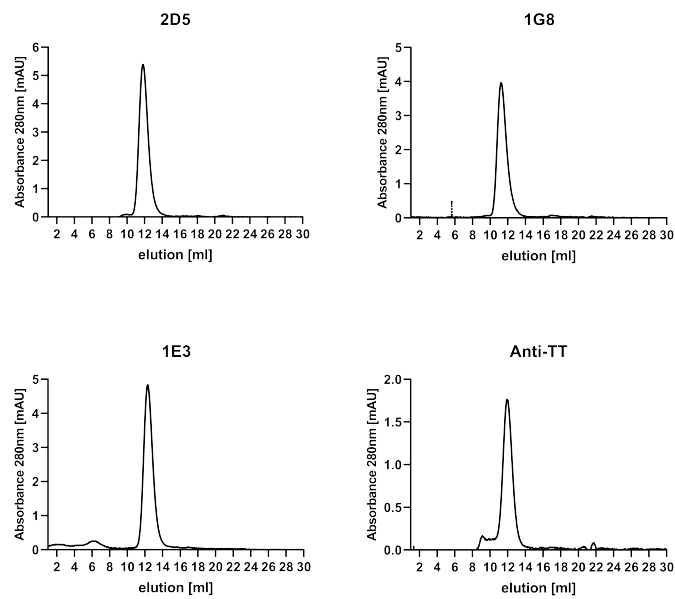

C

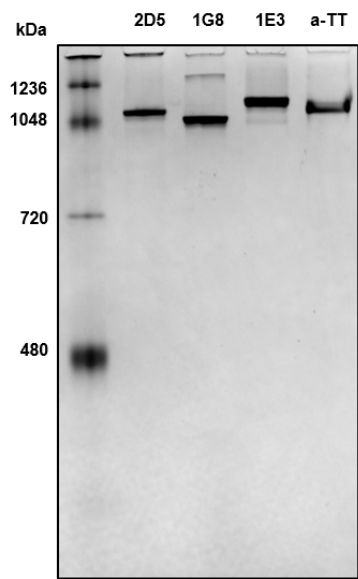

Supplement: Supplementary file 4 — Additional file 4. Figure S3. [file 13075_2021_2609_MOESM4_ESM.pdf]

## TT ELISA

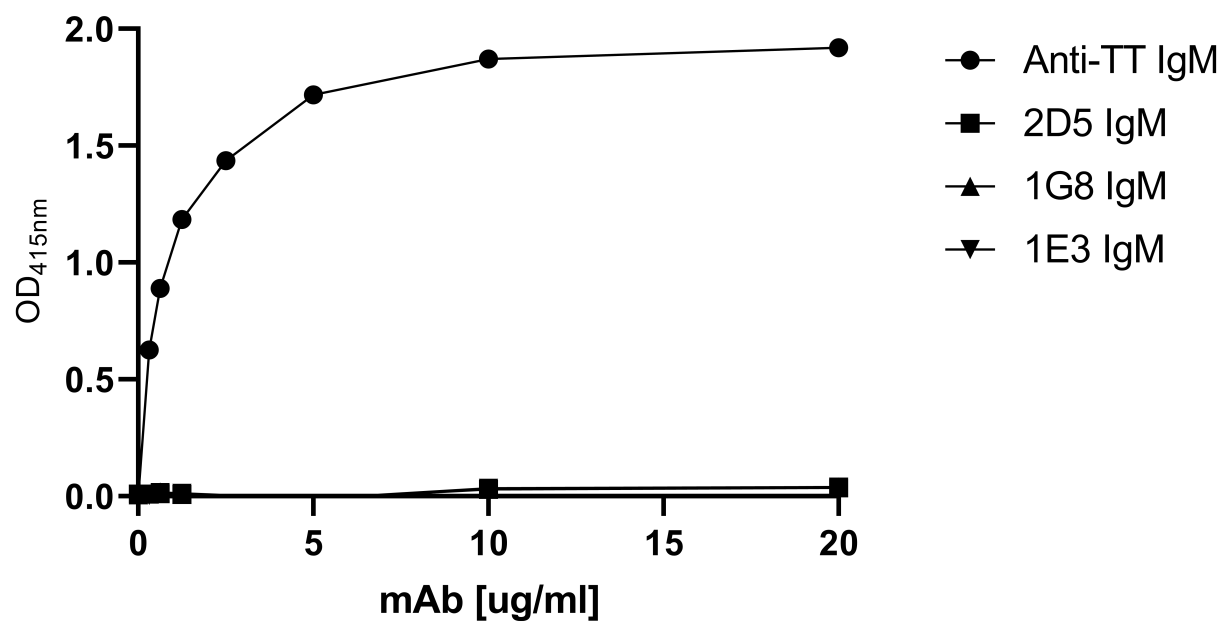

Supplement: Supplementary file 5 — Additional file 5. Figure S4. [file 13075_2021_2609_MOESM5_ESM.pdf]

A

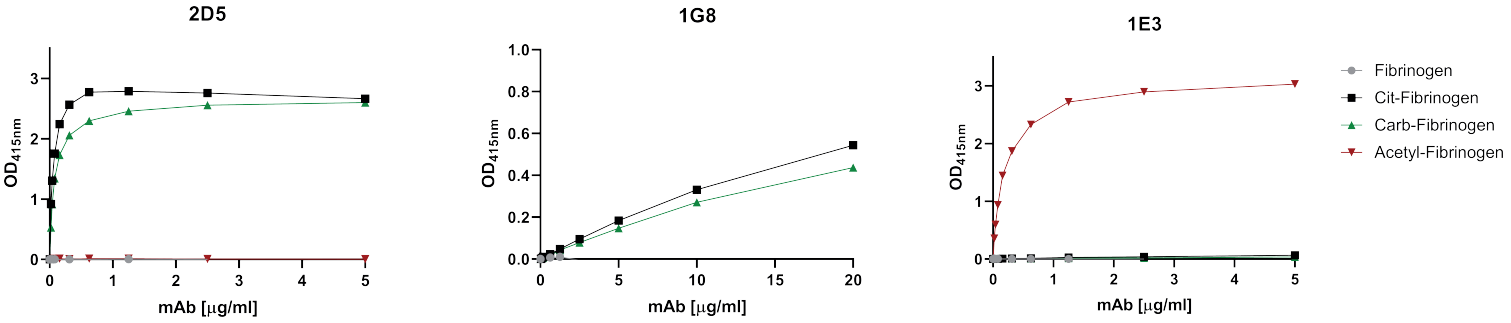

B

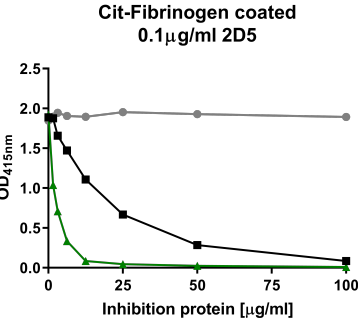

C

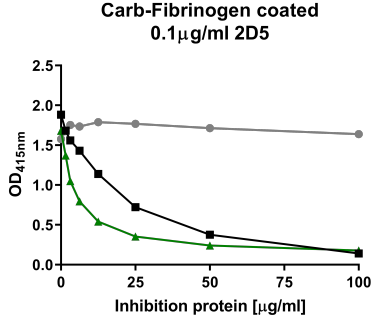

D

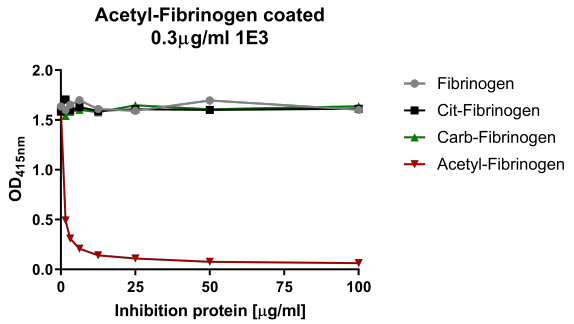

Supplement: Supplementary file 6 — Additional file 6. Figure S5. [file 13075_2021_2609_MOESM6_ESM.pdf]

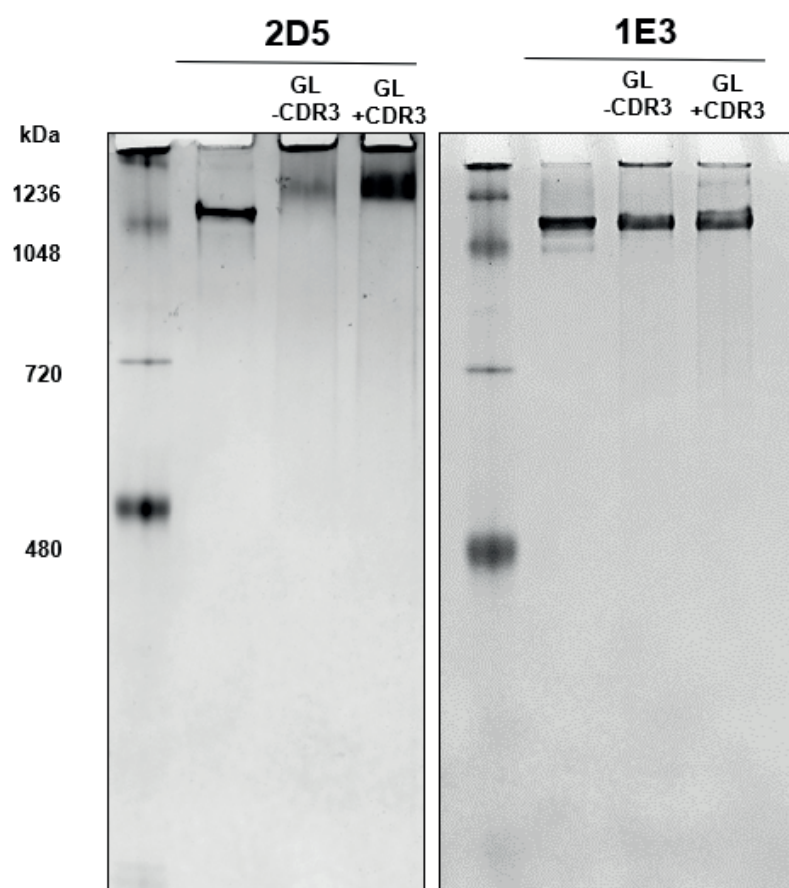

Supplement: Supplementary file 7 — Additional file 7. Figure S6. [file 13075_2021_2609_MOESM7_ESM.pdf]
